# Stakeholder participation in IPBES: connecting local environmental work with global decision making

**DOI:** 10.1080/26395916.2020.1788643

**Published:** 2020-07-13

**Authors:** Cornelia B. Krug, Eleanor Sterling, Timothy Cadman, Jonas Geschke, Paula F. Drummond de Castro, Rainer Schliep, Isimemen Osemwegie, Frank E. Muller-Karger, Tek Maraseni

**Affiliations:** a bioDISCOVERY, Department of Geography, University of Zurich, Zürich, Switzerland; b Center for Biodiversity and Conservation, American Museum of Natural History, New York, NY, USA; c Institute for Ethics, Governance and Law, Griffith University, Brisbane, Australia; d Institute of Plant Sciences, University of Bern, Bern, Switzerland; e Laboratory of Advanced Studies in Journalism – Labjor, University of Campinas, Campinas, Brazil; f Network-Forum for Biodiversity Research, Museum Für Naturkunde Berlin, Leibniz Institute for Evolution and Biodiversity Science, Berlin, Germany, Environmental Information and Communication Services – EIC, Haderslebener Straße, Berlin, Germany; g Center for Development Research, University of Bonn, Bonn, Germany; h College of Marine Science, University of South Florida, Saint Petersburg, FL, USA; i University of Southern Queensland, Toowoomba, Australia, Northwest Institute of Eco-Environment and Resources, Chinese Academy of Sciences, Lanzhou, China

**Keywords:** Patricia Balvanera, Biodiversity conservation, ecosystem services, global collaboration, governance of nature, science-policy interface

## Abstract

The Intergovernmental Science-Policy Platform on Biodiversity and Ecosystem Services(IPBES) strengthens the science-policy interface by producing scientific assessments on biodiversity and ecosystem services to inform policy. IPBES fosters knowledge exchange across disciplines, between researchers and other knowledge holders, practitioners, societal actors and decision makers working at different geographic scales. A number of avenues for participation of stakeholders across the four functions if IPBES exist. Stakeholders come from diverse backgrounds, including Indigenous Peoples and local communities, businesses, and non-governmental organization. They represent multiple sources of information, data, knowledge, and perspectives on biodiversity. Stakeholder engagement in IPBES seeks to 1. communicate, disseminate, and implement the findings of IPBES products; 2. Develop guidelines for biodiversity conservation within member countries; and 3. create linkages between global policy and local actors – all key to the implementation of global agreements on biodiversity. This paper reflects on the role of stakeholders in the first work programme of IPBES (2014–2018). It provides an overview of IPBES processes and products relevant to stakeholders, examines the motivation of stakeholders to engage with IPBES, and explores reflections by the authors (all active participants on the platform) for improved stakeholder engagement and contributions to future work of the platform.

## Introduction

The Intergovernmental Science-Policy Platform on Biodiversity and Ecosystem Services (IPBES) was created in 2012 (Larigauderie 2015; for more details about the early stages of IPBES, see Larigauderie and Mooney 2010), recognizing the need for a science-policy interface for biodiversity (Chapason and van den Hove 2009). IPBES is based on the findings of the Millennium Ecosystem Assessment and modelled after the Intergovernmental Panel on Climate Change (IPCC) (Görg et al. 2010; Koetz et al. 2012). IPBES is an international, intergovernmental body that aims to gather, analyze, and critically evaluate knowledge on biological diversity from various institutions such as national governments and local authorities, universities, scientific organizations, non-governmental organizations, as well as Indigenous Peoples and local communities (IPLCs) (IPBES 2018). It summarizes this knowledge of the status and trends of biological diversity in thematic, regional, and global assessments. To date, the assessments are largely based on reviews of existing scientific literature. IPBES also identifies and addresses the capacity, knowledge, and data needs of its members, experts, and stakeholders; provides policy support through the identification of tools and methodologies relevant for policy, the facilitation of their use, and their further development; and uses a range of communication and outreach tools to ensure a broad outreach and wide impact. As a science-policy interface, IPBES provides an interesting opportunity to explore the nature of the structures and processes of contemporary international environmental governance (Cadman 2011).

IPBES is governed by member delegations representing national governments, but encourages participation of diverse non-state actors, including intergovernmental organizations, international and regional scientific organizations, environment trust funds, IPLCs, non-governmental organizations, and the private sector (UNEP/IPBES 2010) (Figure 2). This builds on lessons learned by the IPCC in its early years. A major goal of having mechanisms for stakeholder engagement is to increase diversity and inclusiveness (Díaz-Reviriego et al. 2019).

Following IPBES’ use of the term, we use ‘stakeholder’ to refer to individuals, institutions, organizations, or groups of people who contribute to, and make use of, the processes and products of IPBES (UNEP/IPBES 2013a, 2014). Various rationales for stakeholder engagement have been put forth in the global policy discourse, including to enhance knowledge, increase relevance, and reduce skepticism about the validity of results (Vohland and Nadim 2015). Stakeholder participation also strengthens the science-policy interface for biodiversity and ecosystem services for the conservation and sustainable use of biodiversity, long-term human well-being, and sustainable development (UNEP/IPBES 2011, p. 7, Annex I.I.). It recognizes other knowledge systems as important elements for understanding nature-people relationships (as an example, see Box 1 on the inclusion of Indigenous and local knowledge (ILK) in IPBES). It improves the effectiveness of the governance of biodiversity (Turnhout et al. 2014; Esguerra et al. 2017; Watson 2018).

Calls for broad participation in intergovernmental processes such as IPBES are rooted in the emphasis on moving away from the exclusive domain of nations in governance of the global commons (Cadman 2011). How stakeholder interactions are coordinated within an institution can have significant bearing on its legitimacy in the eyes of its participants and the public. The values that underpin such interactions include accountability and transparency, but also functional efficacy. Values guide the way in which an institution makes decisions and who is involved in making them. Here, the level of inclusiveness comes into play – such as the opportunities stakeholders have to participate in and contribute to IPBES processes. Effective participation further depends on the resources that participants have at their disposal or that are being made available to them. An institution thus needs to have mechanisms in place to ensure equality of power relations between participants, and seek to encourage behavioral change to create durable solutions to the challenges the institution was set up to address (Cadman et al. 2016).

In this paper, we outline how IPBES has engaged, and is currently engaging with, stakeholders, and reflect on the experiences, perspectives, and opportunities for participation and engagement of multiple social actors in IPBES. To achieve this, we examine the motivation and reasons behind the engagement of stakeholders in IPBES, and explore how the expression of governance values within IPBES impacts stakeholder participation. We further shed light on how stakeholders make use of IPBES outputs and illustrate how IPBES engagement with stakeholders can be improved and the participation of a broad variety of actors can be achieved at regional, national, and local levels.

## Methods

**Figure 1.**
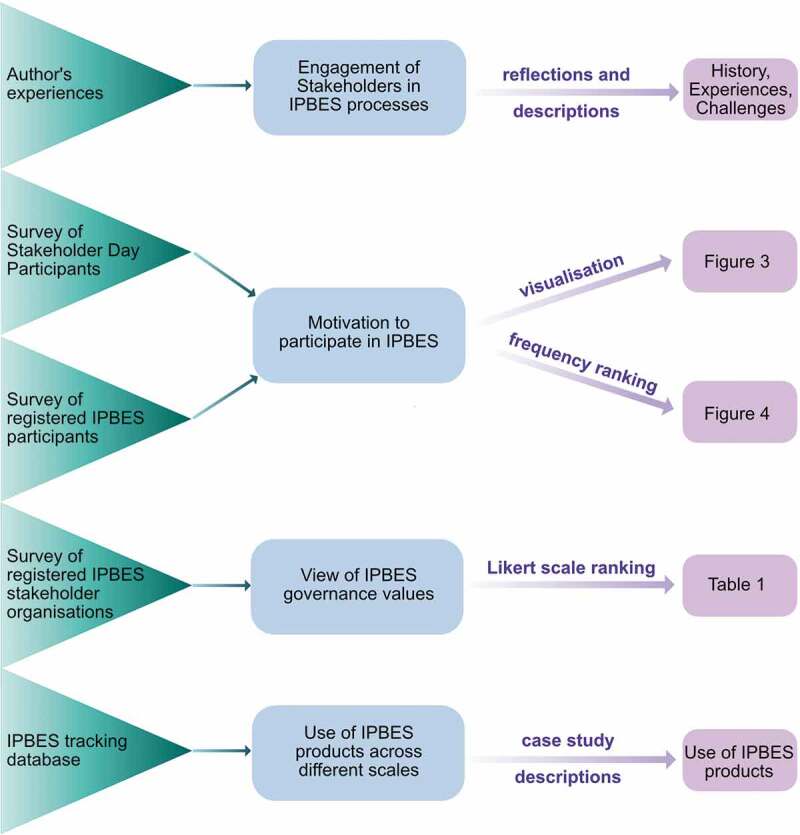
Overview and synthesis of the different sources of information used to address the main questions in this paper, methods and approaches used to answer the key questions, and the sections, figures and tables where results are displayed and discussed.

We employed a variety of methods to explore IPBES’ engagement with stakeholders (Figure 1). The descriptions and reflections emerge from the cumulative and diverse experiences of our engagement as stakeholders in the IPBES process even prior to the formal inception of the platform. We draw on our experiences as members of academic, educational institutions and research networks, as representatives of non-governmental organizations as well as of local conservation agencies; we have contributed to IPBES in many different capacities. This includes participating as observers in all seven IPBES plenaries, as organizers of IPBES Stakeholder Days (events held at the start of IPBES plenary sessions to coordinate stakeholders), founding members of the Open-ended Network of IPBES Stakeholders (ONet, see below for more details), bolstering the assessments, serving as resource people for task forces, and serving as stakeholder coordinators at the subnational, national, regional and global scale for uptake of IPBES products (see Appendix A1 for more details). We further draw on our experiences working with IPLCs as well as on insights from participating in IPLC meetings, e.g. meetings of the International Indigenous Forum on Biodiversity and Ecosystem Services (IIFBES), an IPBES stakeholder network assembling IPLC organizations.

To elucidate the motivations of stakeholders for participation in IPBES plenaries, we conducted a set of interactive stakeholder engagement sessions and a survey of participants during the Stakeholder Day prior to IPBES-7, using the online tool Mentimeter^1^ to engage the participants. Their responses to the questions were visualized in real time and displayed as word-clouds (see Figure 3) as well as in other formats (see Geschke 2019). In order to further understand who was participating in IPBES and what their motivations were, we drew on a survey that was conducted in 2016 by the IPBES Secretariat and IUCN (UNEP/IPBES 2017). The aim of the survey was to understand the motivation behind stakeholder participation and to inform the design of specific outreach activities (UNEP/IPBES 2017). The questionnaire was sent by email to more than 6,300 IPBES stakeholders registered on the webpage at that time. The overall response rate to the survey was 13%. A profile of the respondents is shown in Table A1.

To explore stakeholder views on IPBES governance, we conducted an additional survey of stakeholder organizations between May 6 and 10 May 2019. Survey participants were recruited from the publicly available list of 454 accredited organizations (i.e. accredited as observers in plenaries, and named as such by IPBES) on the IPBES website (https://ipbes.net/accredited-organisations). Thirty-nine organizations responded to the survey, and 29 submitted completed responses. Of the observer organizations, 15 identified themselves as ONet stakeholders. A full list of survey questions is included in Table A3. Respondents were asked to rate IPBES using eleven governance values (inclusiveness, equality, resources, accountability, transparency, democracy, agreement, dispute resolution, behavioral change, problem solving, and durability). Respondents were further asked to rate a number of IPBES components (plenary, work programme, working groups, secretariat, Stakeholder Day, IPBES generally). Each respondent was asked to rate their perception of governance quality via a 5-point Likert scale, from ‘very low’ (1) to ‘very high’ (5). Using an electronic spreadsheet, individual scores of each sector for each indicator were averaged and overall governance quality was determined by adding the average scores of all 11 indicators. As there were 11 indicators, minimum and maximum possible scores were 11 and 55, respectively. Beyond the ratings, respondents were invited to submit comments. The approach adopted here replicates the value-based approach for determining legitimacy, and has previously been used to evaluate stakeholder perceptions of the quality of governance in a number of international environmental agreements and policy instruments (Cadman et al. 2015; Breakey et al. 2017; Glynn et al. 2017).

To complete the picture of IPBES stakeholder engagement, we use cases from the IPBES impact tracking database TRACK^2^ to illustrate how IPBES outputs are used by different stakeholders at different spatial scales (Table A4 in the Appendix section). Further sources of data include peer-reviewed literature on IPBES stakeholders as well as annual or periodic institutional reports summarizing our interactions with IPBES.

**Figure 2.**
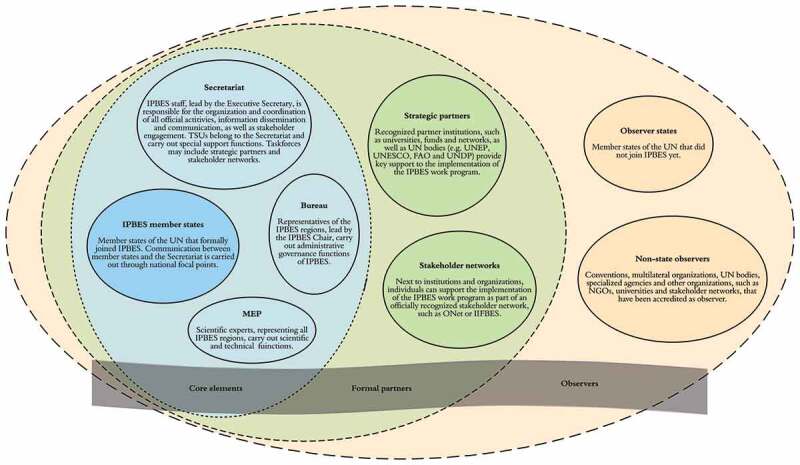
Layers of participation in the Intergovernmental Science-Policy Platform on Biodiversity and Ecosystem Services (IPBES). Left, blue circles: Member states (dark blue) constitute the science-policy platform. The Secretariat including the Technical Support Units (TSUs), the Bureau, and the Multidisciplinary Expert Panel (MEP) (light blue) ensure the administrative, and technical and scientific, functioning of IPBES. Center, green circles: IPBES is supported by partners including UN bodies and stakeholder networks. While stakeholders can contribute to the activities of the work programme, use or benefit from the outcomes of the work programme, and encourage and support the participation of scientists and knowledge holders in the work of IPBES, stakeholders are not entitled to observer status unless they are admitted as such. Right, orange circles: Observer groups include all state members of the United Nations that are not members of IPBES, conventions, multilateral organizations, United Nations bodies and specialized agencies and other organizations that have been approved as observers during previous IPBES sessions. Observers may, upon the invitation of the IPBES Chair, participate in the plenary without the ability to cast votes or join or block consensus.

Our results and discussion follow IPBES in differentiating between member states, which are nation states, and observers (conventions, multilateral organizations, UN bodies, stakeholder-recognized networks, and other organizations that have been accredited as observers https://ipbes.net/about; also see Figure 2).

## Results and discussion

### History and evolution of stakeholder engagement in IPBES

In 2007, members of scientific, governmental, non-governmental organizations, and IPLCs were part of the International Steering Committee of the International Mechanism of Scientific Expertise on Biodiversity (IMoSEB) that initiated and facilitated the process towards the establishment of IPBES (IISD, 2007; Vadrot 2014). In 2010, during the process of developing the foundations of IPBES, mobilization, engagement, and meaningful participation of stakeholders were seen as crucial to ensuring a wide expertise base in the development of IPBES processes and to enhancing the relevance and legitimacy of its deliverables and outputs across different global, regional, and local scales (Larigauderie and Mooney 2010; Vohland and Nadim 2015). Starting in 2012, IPBES established a process for the inclusion of non-member organizations whose representatives may be conferred ‘observer’ status^3^ (UNEP/IPBES 2013b, p. 4), adopted a stakeholder engagement strategy, and established strategic partnerships with stakeholder networks (Decision IPBES-4/4) (UNEP/IPBES 2011).

Similar to member states, accredited non-state observers have the right to deliver statements during the plenary, in particular during the opening and closing sessions. The coordinated groups of stakeholders (belonging or not to the recognized ONet and IIFBES networks) develop such statements in a consultative process that reflect the views and opinions of a broad range of stakeholders. Initially, the rules of IPBES procedures (UNEP/IPBES 2013b) limited the rights of stakeholders to a marginal observer status in the plenary, excluding them from the decision-making process, and restricting them to ‘*support the implementation of the work programme*’ (Granjou et al. 2013; Esguerra et al. 2017). This restriction has been somewhat loosened. In its guide for new observers, ONet remarks that observers can now comment on IPBES plenary agenda items; any proposed position or change, however, must be supported and presented by one or more IPBES member delegation(s). The chair of an IPBES plenary session must then acknowledge these propositions and ask if other delegations agree with them. Only then are these propositions considered in the decision-making process and reflected in the negotiated text. They are recognized only if there is consensual agreement among the member states (Timpte et al. 2018). Only member states are allowed to vote on proposed wording.

Nevertheless, observer organizations have a role to play in expanding the otherwise limited participation of stakeholders in IPBES processes (see e.g. IISD, 2015, p.3; UNEP/IPBES 2019 for examples), and evident in the stakeholder engagement strategy. Stakeholders (acting as observers in the plenary) have further ensured the engagement of observer organizations in the nomination of experts and knowledge holders from different disciplines, knowledge systems, regions, and genders in all of IPBES deliverables (UNEP/IPBES 2018).

### Who is participating in IPBES as a stakeholder and why?

Interest among NGOs in becoming observer organizations has increased over time, in particular in response to the publication of assessments. To date, more than four hundred NGOs and academic institutions are registered with IPBES; a number of new applications from NGOs and organizations from diverse regions of the world are currently underway.

**Figure 3.**
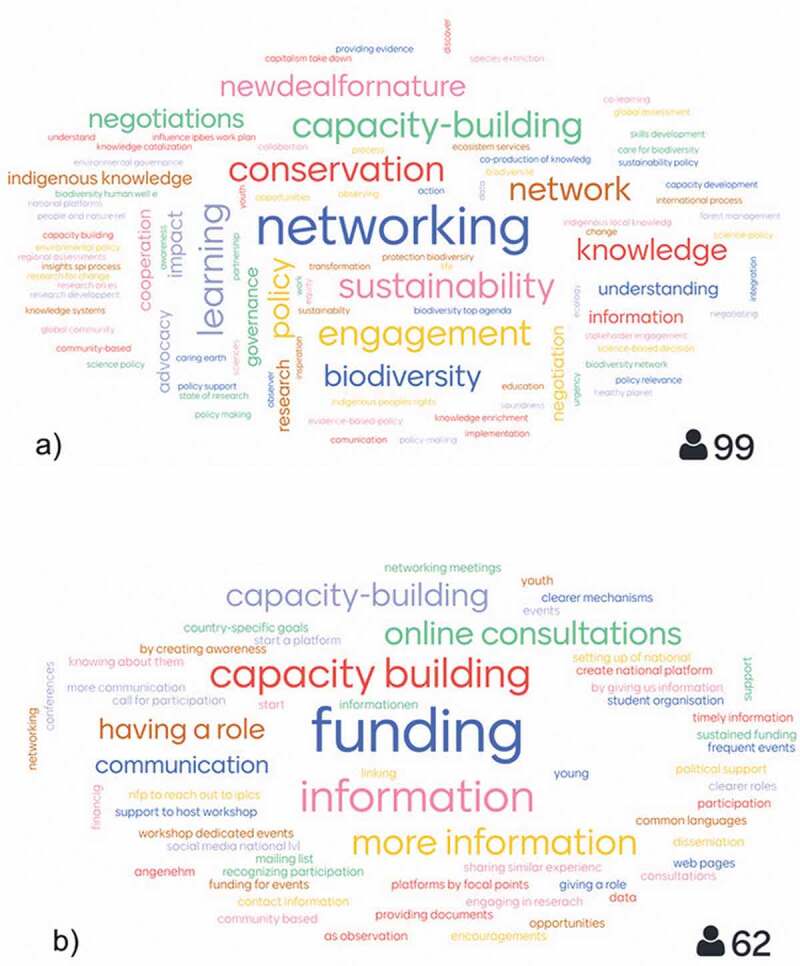
Graphical representation of the results of interactive stakeholder sessions conducted during the IPBES Stakeholder Day 2019 (see Box 2). Specifically, the survey posed the questions a) What is your main interest(s) in participating in IPBES work? and b) How can your participation in national platforms be supported? The number of respondents for each question is listed at the lower right corner (Geschke 2019).

**Figure 4.**
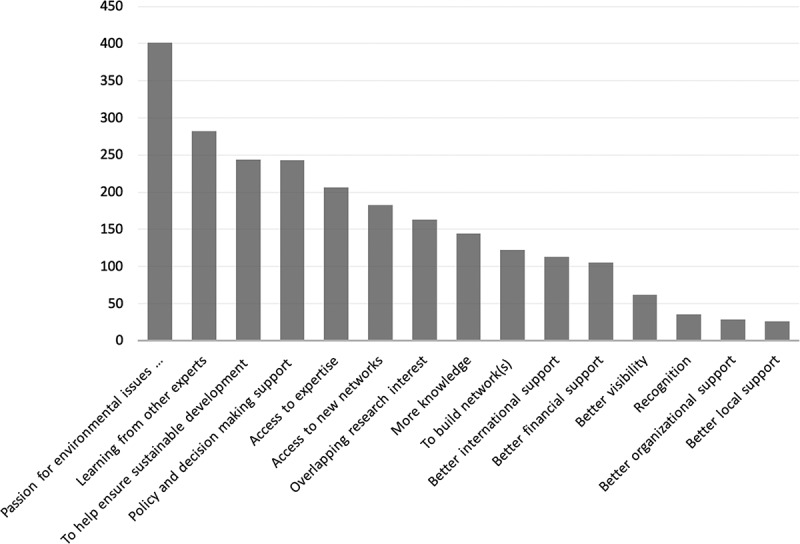
Frequency of responses on the motivation and incentives of observer groups to participate in IPBES. Total number of responses received was 839; survey participants were asked to select up to three response options. Data from the IPBES/IUCN Stakeholder survey conducted in 2016, unpublished and used with permission.

Motivations and incentives to participate in IPBES are highlighted in Figures 3 and 4: Results from the participant survey conducted during the IPBES-7 Stakeholder Day in 2019 show the opportunity for networking, mutual learning, capacity-building, and conservation of nature as the main incentives to participate in IPBES processes and activities (Figure 3). Responses to the 2016 survey varied by region. The motivation most mentioned was ‘Passion for environmental issues and for sustainable use of natural resources’, followed by ‘learning from other experts’, ‘helping to ensure sustainable development’, and ‘policy and decision making support’ (Figure 4). A more detailed breakdown of responses into IPBES regions can be found in Table A2 (UNEP/IPBES 2017).

### Views on the governance of IPBES

The survey reveals differences in views between IPBES observer organizations (observers) and member states (members) (Table 1). Given the small number of survey respondents, and the results being qualitative rather than quantitative, the results presented here are not necessarily representative of IPBES stakeholders as a whole. IPBES member respondents ranked the different institutional elements of IPBES higher than the observers. Observers gave the plenary the lowest overall score for any element (31.9 out of 55). Two respondents who identified themselves as belonging to Indigenous Peoples organizations commented on the plenary. One stated that participation was ‘not effective.’ The other stated, ‘[w]e cannot have adequate time to share [because] we do not have the same rights and privileges as parties (i.e. Member States).’ Conversely, member respondents awarded the plenary and Stakeholder Day(s) the highest score (42.6). Observers also assigned Stakeholder Day(s) the highest overall score (36.9). One observer (researcher) noted that the work programme was ‘geared towards the representation of different stakeholders and integration of different world views.’ However, another observer (NGO) noted that ‘[e]ven on Stakeholders’ Day, the agenda is mostly driven by the IPBES Secretariat.’

**Table 1.** Survey of respondents’ views on IPBES activities (n = 29, May 2019). Fields in light grey are the highest scores per institutional element; the dark grey are the lowest. O: Observers, M: Members.

Institutional Element	Sector	Governance value (Very high – 5; high – 4; medium – 3; low – 2; very low – 1)											Total (out of 55)
		Inclusiveness	Equality	Resources	Accountability	Transparency	Democracy	Agreement	Dispute settlement	Behavior change	Problem solving	Durability	
Plenary	O	2.9	3.1	2.5	3.1	3.2	2.9	2.4	2.6	3.1	2.8	3.3	31.9
	M	4.1	4.0	3.7	3.8	4.2	4.0	4.0	3.7	3.6	3.6	3.9	42.6
Work programme	O	3.6	3.2	2.7	3.2	3.4	3.3	2.8	2.8	3.3	3.2	3.4	34.9
	M	3.6	3.8	3.6	3.7	3.8	3.8	3.9	3.8	3.9	3.6	3.9	41.4
Working groups	O	3.2	3.2	2.5	3.1	3.4	3.1	2.9	2.7	3.4	3.3	3.4	34.2
	M	3.6	3.6	3.4	3.8	3.9	4.0	4.0	4.0	3.7	3.6	3.7	41.3
Secretariat	O	2.8	3.0	2.4	3.1	3.2	3.1	2.8	2.7	2.9	2.9	3.1	32
	M	3.8	3.9	3.9	4.0	3.8	3.9	3.8	3.8	3.7	3.6	3.6	41.8
Stakeholder day	O	3.7	3.4	2.8	3.4	3.6	3.5	3.3	2.9	3.4	3.5	3.4	36.9
	M	4.1	3.7	3.8	3.6	4.1	4.1	4.3	4.0	3.7	3.6	3.6	42.6
IPBES – general	O	3.4	3.2	2.5	3.2	3.6	3.5	2.8	2.7	3.4	3.3	3.4	35
	M	3.8	3.6	3.7	3.7	4.0	3.7	3.8	3.5	3.8	3.9	4.2	41.7

Looking at the individual governance values, observers were mostly well disposed towards the transparency of IPBES in general (3.6 out of 5), but critical observations were nonetheless made. One observer (NGO) pointed out that ‘IPBES itself is not transparent to stakeholders, but holds stakeholders to a very high level of transparency.’ The Stakeholder Day was seen as relatively inclusive, with one respondent (researcher) commenting that ‘the most recent date of [the] Stakeholder Day (28 April 2019) has, in my opinion, achieved a higher level of inclusiveness in terms of actors (e.g., business sector, IPLCs, private sector, platforms engaging in IPBES, etc.) and regions.’ However, the Secretariat received a low rating for inclusiveness (2.8 of 5).

Possibly reflecting their higher level of procedural involvement, IPBES member respondents tended to give higher ratings to decision-making arrangements (4.0 in the case of IPBES working groups, and 4.3 for agreement in the case of Stakeholder Day – overall the highest rating), in particular democracy, dispute settlement, and agreement. There is a discrepancy here with the observers, who provided low ratings for both dispute settlement and agreement in both of the institutional elements surveyed. IPBES member respondents were somewhat negative about IPBES’ capacity to change behavior and resolve problems, and this is where these lower ratings are closer to the views of observer organizations. The most significant point of divergence between the member respondents and observers were the scores attributed to IPBES’s provision of resources (financial, technical, and so forth). Observer organizations rated resources much lower than IPBES member respondents. The lowest rating for resources from observers (and the lowest rating overall) went to the Secretariat, but other elements were also rated poorly by the stakeholder groups. One observer (researcher) recommended that the distribution of resources be:

… more equitable between sectors such as individual scientists and [other] knowledge holders as well as institutions, organizations and different groups working in the field of biodiversity and ecosystem services.

Since it is mostly governments that fund IPBES, it is appropriate to give the perspective of one of the member state respondents:

IPBES has quite limited resources, so distribution of its own resources is limited, but [it] does [make] an effort to do so (see Technical Support Unit[s], etc.). Leveraging the facilitation of other organizations’ resources is not developed yet.

In regard to the durability of the platform, the member respondents appear to be optimistic, giving IPBES a high rating in general. Observers, consistently more cautious, nevertheless rated durability as the highest scoring governance value for the both plenary and working groups. Therefore, there is consensus between observers and member respondents that IPBES has a future moving forward.

### How stakeholders participate in IPBES

The nature and extent of stakeholder participation within IPBES is very diverse. IPBES has developed a suite of channels for the participation of diverse non-state actors, including IPLCs, in the work of the platform. These include, but are not limited to, directly participating in IPBES functions and processes (e.g. in assessments or task forces), participating as observers in the IPBES plenaries or Stakeholder Days, contributing to the assessment reviews, promoting the use of IPBES products by a variety of societal actors (Lundquist et al. 2015), disseminating IPBES messages, capacity building, and enabling contributions to feed IPBES decisions. Stakeholders can contribute data and information that will help to refine, for instance, the predominantly scientific-knowledge based assessments by providing access to other knowledge sources (see Box 1).

Box 1. *Inclusion of Indigenous and Local Knowledge (ILK) in IPBES assessments and processes*

Over the course of the first work programme, Indigenous Peoples and local communities (IPLCs) and their knowledge systems have become increasingly integral to IPBES assessments. Various attempts were made to integrate ILK into the chapters of the Assessment Report on Pollinators, Pollination and Food Production (pollination assessment), one of the first thematic assessments. A collaboration with the Indigenous Peoples of Thailand concludes that understanding the linkages between pollinators and ILK-based management systems is important in areas of high biological diversity managed by ILK holders (IPBES, 2016). This recognition of ILK was an important first step, but in other cases, respectful engagement of knowledge holders outside the academic arena was hampered by the timelines for the assessments and a lack of inclusive processes. In early iterations of the pollination assessment, one of the authors was asked (and declined) to identify ILK holders and request their knowledge on pollination within a five-day turnaround, as this was insufficient time to explain the purpose of IPBES, the use of the information, and to discuss free, prior and informed consent in knowledge sharing processes. For the global assessment, a structured process was established, which engaged IPLCs through consultative dialogues and discussions with specific questions on drivers affecting IPLCs (Garnett et al. 2018).

In addition to IPLC organizations, various institutions such as governments and authorities, universities, scientific organizations, and NGOs have gathered information and conducted analyses and critical evaluation of scholarship on biological diversity as a core activity of IPBES (IPBES 2018). While it was initially difficult for new stakeholders to identify how best to participate in and contribute to the IPBES process, the initiation of Stakeholder Days as well as the establishment of ONet has significantly reduced these concerns (Box 2).

Box 2. *Stakeholder perspectives from IPBES-7 Stakeholder Day, 2019*

In order to elucidate the motivations of stakeholders to participate in IPBES plenaries, we conducted a set of interactive stakeholder engagement sessions during the Stakeholder Day prior to IPBES-7. We used the online tool Mentimeter to engage participants. Their responses to the questions on screen were visualized in real-time word clouds (see Figure 3) and other formats (see Geschke, 2019). According to the results, about half of the respondents identified themselves as coming from the fields of research and education (Geschke, 2019, slide 3). The participants indicated that their main interests in IPBES are networking, capacity-building, conservation and sustainability (Figure 3a) (Geschke, 2019, slide 4). Respondents engage with IPBES in intersessional periods in diverse ways, for example through task force meetings, indigenous and local knowledge dialogues and social media (Geschke, 2019, slides 6-21). One clear gap is the lack of national platforms to serve as exchange hubs in IPBES member states, partly due to lack of funding and capacity building (Figure 3b; Geschke, 2019, slide 30). Respondents also proposed that, in the context of written products, a summary of assessments for educational purposes in plain language would be useful in addition to the existing summary for policymakers. To foster better stakeholder participation, boost the production of IPBES outcomes and improve the uptake of IPBES findings and results, they recommend more transparent information on how to engage in IPBES as well as regional consultations. Respondents offered to contribute to IPBES through assessment reviews, outreach and promoting ideas (Geschke, 2019, slide 34).

To illustrate this, the Stakeholder Days provide an opportunity for a wide range of interested organizations and individuals to obtain updates on IPBES processes, work programmes, the plenary agenda, intersessional activities, and to discuss stakeholder engagement with IPBES. These events are open to all members, observers, and stakeholders of IPBES, so they also provide opportunities to strengthen stakeholder networking. Stakeholder Days began in 2012, and have since been held in advance of each IPBES plenary. In 2016, about 100 stakeholders participated in the IPBES-4 Stakeholder Day (Kuala Lumpur, Malaysia), while 300 stakeholders from all regions of the world participated in the IPBES-7 Stakeholder Day (France, Paris) in 2019. Stakeholder Days are hosted by the IPBES Secretariat and co-organised by ONet and the International Indigenous Forum on Biodiversity and Ecosystem Services (IIFBES) with technical support from the International Union for Conservation of Nature (IUCN) and, in the past, from the International Science Council (ISC).

The outcomes of these meetings included joint statements presented to the plenary and agreed positions on key stakeholder engagement issues. An additional key outcome was the creation of ONet in 2015. ONet is one of the two official IPBES stakeholder networks, along with IIFBES, that organize contributions to IPBES. ONet coordinates IPBES stakeholder’s activities during the intersessional periods (UNEP/IPBES, 2015a, 2015b; ONet, 2019). Any interested party, whether individuals or institutions, is welcome to join ONet, which has 102 registered members (organizations and individuals) as of May 2020.

Stakeholders also interact with IPBES via national-level platforms and through the activities of global-level Technical Support Units (TSUs). The TSUs are part of the Secretariat, designed to support specific IPBES task forces as well as assessments, enabling experts and other stakeholders to participate in the IPBES process. At the time of writing, there were 10 active TSUs around the world (Marquard et al. 2016). TSUs provide a range of services, such as (1) supporting contributions from the community of experts; (2) facilitating capacity building; (3) working with end users of IPBES products; and (4) providing critical discussion of processes taking place on the platform (e.g. discussions about the review of the platform) (https://www.ipbes.net/collaborative-supporters). Some countries have started to develop internal processes to address the inputs of stakeholders to IPBES. For example, in 2015, the Brazilian Platform on Biodiversity and Ecosystem Services (BPBES) was founded via a grassroots approach by a group of scientists in co-production and dialogue with governmental and non-governmental stakeholders (Padgurschi and Joly 2017; Scarano et al. 2019; Pires et al. 2020).

### Experiences of stakeholder use of IPBES products

For many stakeholders, participation in IPBES is important to understand what knowledge and products are required for the IPBES assessment reports, and how this knowledge can be conveyed. Similarly, broad engagement and interaction at and around plenary sessions can help assessment authors to build trust and understanding with knowledge holders outside of academia. The engagement of IPLCs and their knowledge systems is an example of this (Box 1).

The IPBES tracking database (UNEP/IPBES 2019) provides a number of examples regarding how a variety of stakeholders are using IPBES products and outcomes. The pollination assessment played a key role in promoting better pollinator protection practices. The involvement of knowledge holders including Indigenous Peoples in the pollination assessment was crucial to identifying biological and cultural approaches to pollinators and practices for pollinator conservation at national and local scales. Example practices include valuing diversity from cultural and biological standpoints, landscape management practices, and diversified farming systems (Hill et al. 2019).

The IPBES assessment on pollinators resulted in different groups of scientists and NGOs developing strategies to protect pollinators at the national level. For instance, the Polish National Strategy for the Protection of Pollinating Insects includes an analysis of risk factors as well as recommendations for planners, policy-makers, and practitioners (Zych et al. 2018). In Thailand, a dialogue was initiated across Indigenous, local, and scientific knowledge systems, reflecting on the key messages derived from the pollination assessment. The dialogue demonstrated the relevance and possible uptake in policy and practice of transformations of food systems towards sustainability, how biodiversity conservation practices view and engage with IPLCs, and the relationships between science and knowledge systems for ecosystem governance (Mai and Rai 2019).

A range of organizations, many of them IPBES stakeholders (BIP, 2019), are both users and contributors of relevant data and indicators used in IPBES assessments. They answer the need for developing and delivering biodiversity indicators for IPBES and other biodiversity-related conventions by establishing baselines and conducting evaluations of biodiversity and ecosystem change. An example is the Group on Earth Observations Biodiversity Observation Network (GEO BON). Here, several groups are collaborating to develop biodiversity and environmental observing frameworks and the relevant scientific data synthesis. Much of this process centers on the definition of key variables, termed Essential Biodiversity Variables (EBVs), coming from science and other knowledge systems, and the establishment of focus themes such as the Marine Biodiversity Observation Network (MBON). MBON works closely with UNESCO to provide guidelines on marine biodiversity. In this context, observatories are vehicles to develop better coordination between scientists and other stakeholders, including IPBES and national practitioners (Muller-Karger et al. 2018; Miloslavich et al. 2018; Canonico et al. 2019).

IPBES and its stakeholders can provide scientific and community science (Charles et al. 2020) with specific requirements about indicators useful for assessments. IPBES also could promote wide use of these networks, observing groups, and capacity building efforts (Bax et al. 2018; Benson et al. 2018) to build a community of practice for observations and information management and applications. In this way, major groups of stakeholders will be integral to the development and implementation of IPBES work programmes with a focus of activity in specific areas of action such as NGOs, business and industry, volunteers, IPLCs, and farmers. There are already experiences with citizen observatories at national and international levels that gather information and evidence on sustainability practices from diverse parts of the world (e.g. https://www.conservationevidence.com).

The IPBES conceptual framework (Díaz et al. 2015) has also been useful in other contexts such as medicine, where the One Health concept encompasses the IPBES conceptual framework, recognizing that emerging infectious diseases share similarities with biological invasions, and that ecosystem change has an impact on human well-being (MHN, 2019). Disease outbreaks, such as the current COVID-19 pandemic, are a direct result of human activities impacting the planet, illustrating the need to adopt the One Health approach as part of all decision-making (Settele et al. 2020).

These examples demonstrate how stakeholder engagement raises awareness, catalyses knowledge generation, supports capacity building, and informs policy making.

### Challenges in stakeholder engagement in IPBES

Scientists and other knowledge holders find participating in the IPBES process challenging for a variety of reasons (Vohland and Nadim 2015; Hallosserie 2016; Schliep and Vohland 2017). Time constraints are seen as the main barrier to the participation of experts in IPBES, followed by a lack of time remuneration and lack of support from current employment. The decision-making and communication structures of IPBES are perceived as unclear and inefficient. This is due, *inter alia*, to judgements that the facilitation of access to information and data by the TSUs leaves room for improvement (Schliep and Vohland 2017). The desired disciplinary balance between academic and non-academic experts has also not been achieved in the IPBES working groups (i.e. groups formed to accomplish IPBES functions, coordinated by the Secretariat). Geographical balance is another serious bias that is constantly challenging the success of IPBES (e.g. Kovács and Pataki 2016).

The integration of IPLCs and ILK in IPBES processes is a contentious issue. At the global policy level, the position has been very clear: Indigenous processes are a national (domestic) issue, and within IPBES it is still difficult to speak about the rights of Indigenous Peoples to their territories and to self-determination. While IPBES products recognize the role Indigenous Peoples have played in biodiversity conservation and that biodiversity appears to be faring better in Indigenous lands than elsewhere (UNEP/IPBES 2019a), this recognition seems to be disconnected from the assessment production process, given the multiple calls for having local knowledge experts (usually academics focused on ILK), with very few calls for the participation of knowledge holders themselves (IPLCs). Additionally, the volunteer nature of authorship, coupled with high expectations for self-funding to attend multiple international meetings annually, can preclude effective IPLC participation in assessments.

Participation of stakeholders from lower-income countries is further hampered by a lack of transparency in the observer admission process. Although IPBES has established a procedure for the admission of observers that sets out time limits and information required for an application (UNEP/IPBES 2014), it does not formally accept a quarter of observer applicants. These applicants are not provided the rationale for the refusal. As a result, mainly stakeholders from European institutions or international organizations lead the stakeholder processes.

To enhance broad participation in IPBES, and increase diversity of stakeholders, processes for stakeholder engagement in IPBES need to be transparent, and entry points for stakeholder engagement need to be clearly defined. For example, the success of applications to receive observer status can be increased by enhancing transparency in the review of application. This allows potential observers to prepare improved applications and provide information that is relevant for the application.

Financial resources and supportive structures are needed to enhance the ability of professional scientists, IPLCs, and other experts to participate in IPBES processes at national level as well as the international level, and to close gaps in geographical, disciplinary, and other representation (Marquard et al. 2016).

Capacity building is crucial in enhancing effective participation in IPBES. The IPBES Fellowship programme engages early career researchers in assessments and in capacity development workshops. The fellowship provides them with mentorship opportunities and equips them with skills and capacities to take on leadership roles in IPBES processes and in their home countries. In this way, the leadership in IPBES will be gradually transferred from WEOG to other regions (Gustafsson et al. 2020).

## Conclusions and outlook

The work of IPBES is considered authoritative and of high quality (e.g., Potts et al. 2016; Kovács et al. 2017; Pascual et al. 2017; Rosa et al. 2017; Hill et al. 2019). Stakeholders are engaged via a grassroots efforts that contribute to IPBES assessments and facilitate the uptake and implementation of IPBES products. For example, the organization and facilitation of expert dialogues and other capacity-building activities are consolidated by stakeholder networks, specifically the Open-Ended Network of IPBES Stakeholders (ONet) and the International Indigenous Forum on Biodiversity and Ecosystem Services (IIFBES).^4^ Achieving the desired disciplinary balance between academic and non-academic experts in IPBES task forces, working groups and assessments, as well as striving towards geographical balance will contribute greatly to IPBES success and credibility, and motivate stakeholders to participate in the work of the platform.

In order to succeed, the governance of IPBES, similar to the development of policy options and transformation measures towards sustainability, needs to be tailored to and built from local knowledge systems (e.g. experiences like Sawhney et al. 2007). Collaboration with Indigenous and Local Knowledge (ILK) holders through participatory approaches can provide a channel for honoring diversity and productive engagement with knowledge holders in other sectors. Prioritizing engagement with ILK produces a best practice model and policy for respectful and collaborative engagement (Hill et al. 2020). Encouraging the exchange of knowledge between a range of different knowledge holders can facilitate the transfer of experience where similar sustainability challenges arise. Policy action always is local, as is the case with field research, and needs to be sensitive to local conditions. Effective dialogue will require the building of mutual trust and confidence between ILK holders and non-local natural and social scientists through cultural respect and sensitivity. Improving knowledge exchange at the local level will enhance the effectiveness of the implementation of the IPBES work programme, which strives to achieve sustainable development in line with the 2030 agenda and the Sustainable Development Goals (SDGs), while protecting and restoring biodiversity (SDG-UN, 2019; Solberg 2019).

Continued stakeholder engagement would be enhanced by attending to financial and communication challenges. Expert engagement in IPBES activities can improve by properly recognizing their voluntary activity. It is important to encourage academics as well as other knowledge holders, including IPLCs and other practitioners, to participate, in order to produce comprehensive documents that include broader knowledge on biodiversity and nature. This represents a more inclusive process and can lead IPBES to be clearly meaningful for a broader range of actors at the national and subnational levels. IPBES should develop stronger links to organized scientific stakeholder groups, the private sector, organized civil society, the education sector, as well as IPLC networks, to develop more accurate and usable assessments.

From a financial perspective, the IPBES members have prioritized environmental assessments for budget allocation (Brooks et al. 2014). The IPBES budget available for stakeholder engagement is small, leading to resource challenges, dissatisfaction among some stakeholders, and inequity in stakeholder access to IPBES processes and outputs. Most activities rely on alternative sources of funding, for example, via national science-policy platforms, which themselves can promote transdisciplinary knowledge exchange (Geschke et al. 2020) but face financial problems (Marquard et al. 2016). Meaningful and equitable participation of stakeholders will involve further attention and resources allocated to the implementation of the stakeholder engagement strategy and to emerging areas of concern of these communities of interest (Klenk et al. 2015). The low rating for inclusiveness in the survey should encourage the IPBES Secretariat to identify ways to involve observer organizations. Issues such as dispute settlement and reaching agreements among participants require additional involvement of observers. Marginal participation from different regions of the world should be addressed by including financial support as well as better and transparent accreditation criteria, to properly address the underrepresentation of groups outside the WEOG region. Effective stakeholder engagement will involve opening IPBES decisions to nurturing feedback. Effectively recognizing that social actors other than governments have more relevant roles to play in biodiversity conservation than just receiving information would be a major first step.

## Supplementary Material

Supplemental Material

## Notes

1. https://www.mentimeter.com, Mentimeter AB, Alströmergatan 22 SE-112 47 Stockholm Sweden.

2. https://ipbes.net/impact-tracking-view.

3.
United Nations bodies and specialized agencies, intergovernmental organizations and secretariats of conventions; IPBES bodies; non-governmental organisations, business/industry organisations, education and research institutes and organisations and other types of organisations. https://www.ipbes.net/accredited-organisations.

4. www.ipbes.net/stakeholder-events.
