# Supplementary material for: Stakeholder participation in IPBES: connecting local environmental work with global decision making
Source: Ecosyst People (Abingdon). 2020 Jul 13;16(1):197–211. doi: 10.1080/26395916.2020.1788643 (PMC7484931; doi:10.1080/26395916.2020.1788643)
Supplement: Supplemental Material [file TBSM_A_1788643_SM7352.pdf]

APPENDIX

Appendix 1: Authors's roles in IPBES

Collectively, the authors have participated as non-state observers (representing different academic and research institutions and NGOs) in all seven IPBES plenary sessions, were part of the organizing team as well as participants of the Stakeholder days, participated in the parallel meetings of Indigenous Peoples and Local Communities (IPLC) during the IPBES plenaries, and were founding members of the Open-ended Network of IPBES Stakeholders (Onet) initiative. In addition, the authors made contributions themselves, and/or have mobilized and supported contributions from others, to the review of various IPBES assessments, including the recently released Global Assessment report; the regional assessment reports; Scenarios and models; Pollinators, pollination and food production; and the ongoing Values and upcoming Nexus assessment. Additional functions undertaken in IPBES include serving as resource persons in the task force on knowledge and data, and contributing, as part of their current job/institutions, to facilitate contributions from West African stakeholders (WABES project), IPLCs, and other scientists to IPBES work; awareness-raising and dissemination of IPBES products; collection of relevant biodiversity data, and generation of syntheses (e.g. as members of the Brazilian Platform on Biodiversity and Ecosystem Services – BPBES, GEOBON or Future Earth) and as a co-chair of the MBON- Marine Biodiversity Observation Network.

Table A1. Profile of the respondents of the survey conducted by IPBES Secretariat and IUCN 2016. Used with permission from UNEP/IPBES, 2017.

**Characteristics of respondents**	**Main results (n= 839)**	
• Gender (%)	Men 63, Women 37	
• Region of the world (%)	Africa (AF) 23<br>Asia –Pacific (AP) 20<br>Eastern Europe (EE) 7	Latin American and Caribbean (LAC) 19<br>Western Europe and Other States (WEOG) 29<br>NA 2
• Disciplinary profile (%)	Natural Sciences 46<br>Social Sciences 16<br>Other disciplines 6	Other Knowledge System 2<br>NA 30

Table A2. Percentage of responses for each IPBES region for motivation and incentives for participating in IPBES (Adapted from UNEP/IPBES, 2017, Figure 42). Participants were asked to select up to three options in this section of the survey.

**Region**	**Africa**	**Asia-Pacific**	**Eastern Europe**	**Latin American and Caribbean**	**Western Europe and Other States**
Access to expertise	26%	19%	10%	24%	20%
Access to new networks	23%	16%	8%	22%	29%
Better financial support	33%	29%	4%	20%	11%
Better international support	27%	28%	5%	23%	17%
Better local support	31%	12%	12%	27%	19%
Better organizational support	52%	28%	7%	7%	3%
Better visibility	21%	18%	3%	11%	44%
Learning from other experts	24%	19%	8%	21%	28%
More knowledge	15%	27%	10%	15%	30%
Overlapping research interest	17%	19%	7%	12%	42%
Passion for environmental issues and for sustainable use of natural resources	29%	18%	7%	16%	29%
Policy and decision making support	13%	20%	7%	22%	39%
Recognition	9%	46%	0%	14%	31%
To build network(s)	17%	18%	6%	17%	39%
To help ensure sustainable development	22%	17%	5%	26%	27%
Other	6%	11%	0%	6%	67%

Table A3. List of survey questions about governance in IPBES (May 2019) excluding introductory materials and questions regarding respondent demographics.

Governance value	Questions
**Inclusiveness**	Do you think IPBES facilitates inclusiveness among stakeholders? What rating would you give?
**Equality**	Do you think IPBES facilitates equality among stakeholders? What rating would you give?
**Resources**	Do you think IPBES facilitates the distribution of resources (financial, technical, etc.) among stakeholders? What rating would you give?
**Accountability**	Do you think IPBES facilitates accountability among stakeholders? What rating would you give?
**Transparency**	Do you think IPBES facilitates transparency among stakeholders? What rating would you give?
**Democracy**	Do you think IPBES facilitates democracy among stakeholders? What rating would you give?
**Agreement**	Do you think IPBES facilitates the making of agreements among stakeholders? What rating would you give?
**Dispute settlement**	Do you think IPBES facilitates the settling of disputes among stakeholders? What rating would you give?
**Behavior change**	Do you think IPBES facilitates behavior change regarding biodiversity and ecosystem services? What rating would you give?
**Problem solving**	Do you think IPBES facilitates problem solving regarding biodiversity and ecosystem services? What rating would you give?
**Durability**	Do you think IPBES facilitates durable outcomes regarding biodiversity and ecosystem services? What rating would you give?

Table A4. Cases displaying the use of IPBES outputs by different stakeholders

Case	Aim	Outcome	Stakeholder involvement	Scale of impact
IPBES Conceptual Framework Used to Analyze Biocultural Approaches to Pollinator Conservation by Indigenous People and Local Communities	To analyze the biocultural approaches to pollinator conservation by IPLCs globally by using the Conceptual Framework of the IPBES.	Analysis identified practices useful for pollinator conservation, grouped into three categories: cultural and biological valuation of biodiversity; landscape management practices; and diversified farming systems.	Scientists, IPLCs	Global<br>National<br>Local
National strategy to protect pollinating insects in Poland prepared on the basis of IPBES Assessment.	Concerned Polish scientists and Greenpeace prepared a national strategy to protect pollinating insects in Poland prepared on the basis of IPBES Assessment.	Strategy aimed at protecting pollinators In Poland.	Scientists, NGOs.	National
Dialogue across indigenous, local and scientific knowledge systems.	Reflection of the key messages derived from the Pollination Assessment in order to analyse, adapt, and interpret the key messages derived from the Pollination Assessment.	Identification of instruments and practices for policy makers such as transformations of food systems towards sustainability; how biodiversity conservation practices views and engages with IPLC*s; relationships between knowledge systems for ecosystem governance towards respect and collaboration.	Holders of indigenous knowledge, scientists, NGOs.	Local
Brazilian Platform on Biodiversity and Ecosystem Services (BPBES), a national, but non-state platform	To produce regular syntheses of academic science and traditional knowledge on biodiversity, ecosystem services and their relations with human well-being.	Summary for Policy Makers and Reports: the Brazilian assessment on Biodiversity and Ecosystem Assessment, and Thematic reports on pollination; restoration; water; climate change and ILK	Mostly scientists, but state and non-state (ILK-holders, private sector, journalists, NGO) were consulted	National

IPBES Conceptual Framework used in medical publication to illustrate interactions between human, animals and environment in emerging infectious diseases and biological invasions.	To analyse Nature's Contributions to People (NCP) for demonstrating that emerging infectious diseases and biological invasions have an impact on human well-being.	Tools, methods and activities were identified to facilitate objectives of reducing impacts of the etiological agents.	Doctors and health system users	Local
BIP[a] is a global initiative to promote the development and delivery of biodiversity indicators.	BIP launches data visualization platform to promote and coordinate development and delivery of biodiversity indicators for use by IPBES and other biodiversity-related conventions.	Categories of indicators according to geographical location, habitat type, policy and conservation actions, sustainable use of natural resources and land, etc.	Stakeholders played a role as direct users of the indicators as well as refining and testing them.	Global<br>National<br>Local
WABES[b] is a capacity building initiative to support the contributions of ECOWAS West African countries to IPBES work programmes	WABES organized a regional workshop to highlight information on IPBES Africa regional Assessment, and provide guidelines on its uptake in national policies	Reflection of key messages in national realities, roadmaps for its uptake, draft national implementation plan, and co-development of research topics to address policy-relevant knowledge gaps including land degradation and the food-water-livelihood nexus.	State and non-state actors (scientists, NGOs, practitioners & ILK-holders)	Regional<br>National

[a]Biodiversity Indicators Partnership. [b] WABES supports the contribution of West Africa to IPBES.
